# Sense of agency in joint action: a critical review of we-agency

**DOI:** 10.3389/fpsyg.2024.1331084

**Published:** 2024-01-31

**Authors:** Alexis Le Besnerais, James W. Moore, Bruno Berberian, Ouriel Grynszpan

**Affiliations:** ^1^Information Processing and Systems, Office National d’Etudes et Recherches Aérospatiales, Salon de Provence, France; ^2^Laboratoire Interdisciplinaire des Sciences du Numérique, CNRS, Université Paris-Saclay, Orsay, France; ^3^Psychology Department, Goldsmiths University of London, London, United Kingdom

**Keywords:** sense of agency, collective identity, joint action, intentional binding, responsibility, human-machine interaction

## Abstract

The sense of agency refers to the experience of control over voluntary actions and their effects. There is growing interest in the notion of we-agency, whereby individual sense of agency is supplanted by a collective agentic experience. The existence of this unique agentic state would have profound implications for human responsibility, and, as such, warrants further scrutiny. In this paper, we review the concept of we-agency and examine whether evidence supports it. We argue that this concept entails multiplying hypothetical agentic states associated with joint action, thus ending up with an entangled phenomenology that appears somewhat speculative when weighted against the available evidence. In light of this, we suggest that the concept of we-agency should be abandoned in favor of a more parsimonious framework for the sense of agency in joint action.

## 1 The emerging concept of we-agency

The sense of agency refers to the experience of control over one’s actions and their effects in the environment (Haggard and Chambon, 2012). In recent years, there has been an increased focus on the social factors that influence this experience (see reviews Silver et al., 2021; Loehr, 2022; Zapparoli et al., 2022). A key claim is that social factors can lead to a fundamental change in the nature of the sense of agency, from self-agency to we-agency. We-agency has been conceptualized as the merging of the agentic identities of individuals who are performing an action together. The notion of we-agency is important given the close link between agency and responsibility (Frith, 2014): The sense of agency is considered a key element when it comes to distinguishing our actions from those of others and, as such, it is a necessary condition to personal liability. If the sense of agency can indeed shift from an individual to a collective identity, what are the consequences for individual (and collective) responsibility? Furthermore, the notion of we-agency is also timely in light of current trends in digital technology, the result of which is a rapid change in the nature and scope of human-machine interaction. Indeed, recent technological developments allow users to interact with autonomous agents that can potentially become actual teammates (McNeese et al., 2018; Wynne and Lyons, 2018). How we experience agency when we cooperate with artificial agents thus becomes critical.

A number of empirical studies investigated the sense of agency when two individuals cooperate. They operationalized joint action in tasks involving co-manipulation of a device (Obhi and Hall, 2011a; van der Wel et al., 2012; Dewey et al., 2014; van der Wel, 2015; Grynszpan et al., 2019; Cho et al., 2020; Le Bars et al., 2020; Jenkins et al., 2021) or turn-taking (Bolt et al., 2016; Bolt and Loehr, 2017, 2021; Loehr, 2018; Sahaï et al., 2019, 2022; Hayashida et al., 2021; Shiraishi and Shimada, 2021). For instance, in the study by Grynszpan et al. (2019), two co-actors co-manipulated connected haptic handles enabling them to feel each other’s forces. Co-actors could also have complementary roles as in an experiment (Jenkins et al., 2021) where one co-actor moved a mouse toward a target, while the other clicked when the target was reached. Turn-taking tasks required co-actors to alternate in producing tones (Bolt et al., 2016; Bolt and Loehr, 2017, 2021; Loehr, 2018) or respond to different stimuli (Sahaï et al., 2019, 2022). Some additional studies investigated hierarchically structured tasks where the action of a leader triggers an action from a follower (Weiss et al., 2011; Pfister et al., 2014; Capozzi et al., 2016; Caspar et al., 2020).

In the following, we first review evidence of changes in the sense of agency when individuals perform joint actions with others. Joint action refers to situations where two or more agents are collaboratively working together to bring about change in their environment (Sebanz et al., 2006). This definition is broadly interpreted in the present article, that includes hierarchically structured joint actions where some agents command others. Second, we summarize how various researchers conceive these changes and examine the notion of we-agency. We suggest that the evidence is currently lacking and outline an approach that may provide more clarity on this issue.

## 2 Evidence for change in the sense of agency in the context of joint action

**Figure 1:**
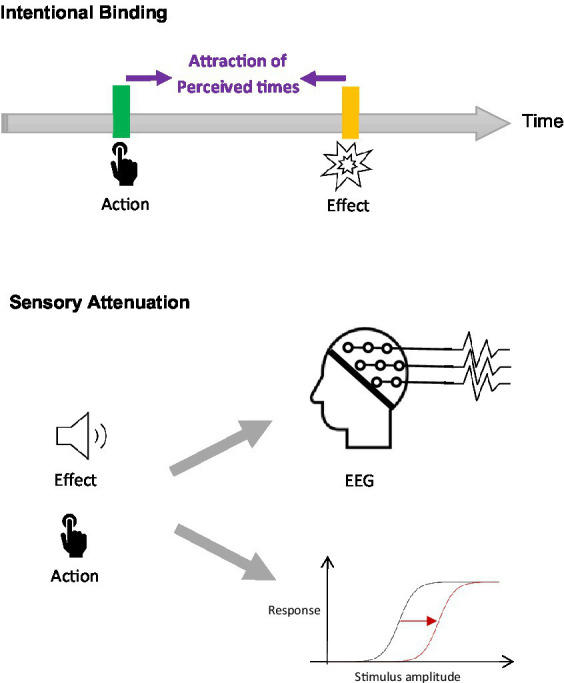
Methods used to measure the implicit sense of agency. The upper panel represents Intentional Binding, which denotes a phenomenon of attraction between the perceived timings of the action and its effect. The lower panel represents sensory attenuation, that is, the perceived attenuation of the sensory feedback of action. Sensory attenuation can be measured using EEG or participants’ verbal responses. The latter is symbolized by the displacement of a logistic function: In a typical experimental paradigm measuring sensory attenuation with verbal responses, participants are asked which are the loudest between a tone stimulus and comparison tones with varied amplitudes. Their responses are plotted against comparison amplitudes and fitted with a logistic function. The displacement of the logistic function when the tone stimulus is self-generated provides a measure of sensory attenuation.

Measuring the sense of agency relies on a variety of processes that involve different levels of awareness (Synofzik et al., 2008). The explicit level is associated with judgments of agency, which tap into reflexive reasoning. It is usually measured by asking participants to report their feeling of control or authorship over actions. The implicit level depends to a greater degree on sensorimotor monitoring mechanisms that can be evidenced using indirect perceptual effects such as sensory attenuation and intentional binding (Haggard, 2017) (Figure 1). Other perceptual effects have been proposed as markers of agency (Le Besnerais et al., 2023) but their usage by the wider research community is still limited. The impact of joint action is outlined in this section beginning with the implicit level and then the explicit level.

The most convincing evidence at the implicit level for a change in the sense of agency during joint action stems from the Intentional Binding effect. Intentional Binding is a phenomenon whereby a voluntary action is experienced as happening later in time and its effect as occurring earlier compared to when the action is not intentional or when there is no action-effect causal relationship (Haggard, 2017). For the last two decades, intentional binding has been accepted as a typical metric of the sense of agency (Moore and Obhi, 2012). Interestingly, several studies revealed that Intentional Binding was also present for the co-actor’s action during joint action and not only for one’s own (Obhi and Hall, 2011a; Grynszpan et al., 2019; Sahaï et al., 2019, 2022; Hayashida et al., 2021; Jenkins et al., 2021). In a seminal study (Obhi and Hall, 2011a), pairs of participants pressed a spacebar together, which triggered a tone after a delay. The time estimates between the keypress and the tone did not differ whether participants reported pressing the spacebar before or after their partner, thus suggesting that the Intentional Binding effect experienced by participants was as effective when their partner initiated action than when they themselves initiated it. It is worth mentioning that the Intentional Binding effect can also occur when one is simply observing the action of another agent without being embedded in joint action (Poonian and Cunnington, 2013; Suzuki et al., 2019; Roselli et al., 2021). This coexistence of Intentional Binding for self and other generated actions was confirmed by a meta-analysis (Zapparoli et al., 2022). It is noteworthy that this meta-analysis reported higher magnitudes of Intentional Binding for self-produced actions compared to other-produced actions. However, this difference may have been driven by some of the included studies in which participants were merely observing another agent without being involved in any joint action. Experiments on joint action have also evaluated the sense of agency using sensory attenuation, another typical method used to measure the Sense of Agency, which refers to the perceived attenuation of sensory feedback resulting from self-generated actions and can be assessed using behavioral measures or EEG (e.g., Bolt and Loehr, 2021) (Figure 1). Yet, mixed results emerged from these investigations (Weiss et al., 2011; Loehr, 2013; Bolt and Loehr, 2021).

When it comes to the effect of social context on the explicit level of agentic experience, a common approach is to ask participants to rate their personal contribution or control over the outcome of joint action (van der Wel et al., 2012; Dewey et al., 2014; van der Wel, 2015; Grynszpan et al., 2019). Such studies have indicated that participants’ responses are consistent with their role and the amount of physical force they put into the joint action. When co-actors had complementary roles, their ratings of control increased (Dewey et al., 2014; van der Wel, 2015) and could even be inflated (van der Wel et al., 2012). Another line of research probed the collective nature of joint agency with scales ranging from shared to independent control (Bolt et al., 2016; Bolt and Loehr, 2017; Loehr, 2018; Shiraishi and Shimada, 2021). Increased coordination between partners (Bolt et al., 2016), the predictability of the partner (Bolt and Loehr, 2017), and success in joint performance (Loehr, 2018) tended to orient those ratings toward shared control. In a nutshell, findings based on explicit ratings of agency suggest a qualitative shift of the sense of agency toward a shared experience in the context of joint action. This adds to the changes observed in the implicit level with the Intentional Binding effect. However, the specific nature of this change has yet to be clarified - this is something we turn our attention to in the next section.

## 3 Characterizations of the sense of joint agency

**Figure 2:**
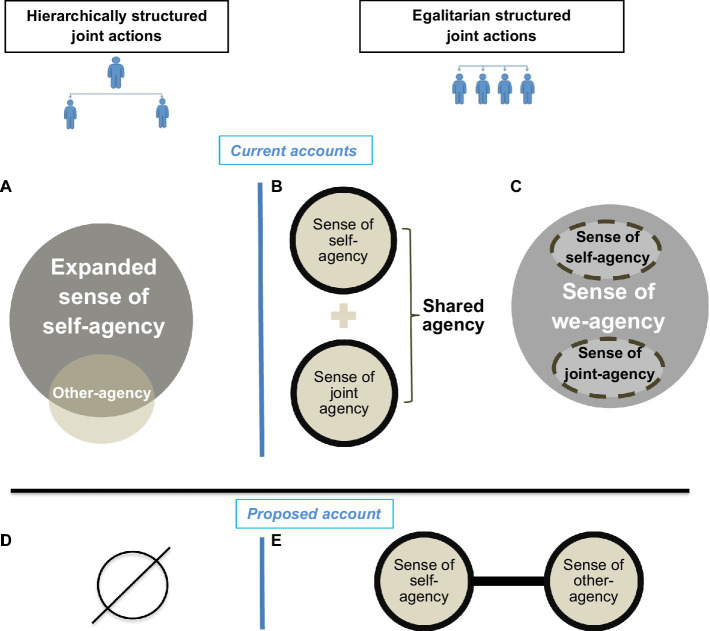
Current and proposed accounts of the different hypothetical agentic states emerging in the context of joint action. The upper panel displays the current accounts retrieved from the literature: **(A)** Expansion of self-agency would occur in the leader of hierarchically structured joint actions. **(B)** Shared agency and **(C)** we-agency would occur in the context of egalitarian joint actions. Sense of self-agency would remain in the former, while it would be dissolved in the latter. Dashed lines: boundaries lost; Solid lines: boundaries present; The lower panel displays the accounts we propose: **(D)** Loss of sense of agency in the context of hierarchically structured joint actions, **(E)** Sense of joint agencies: Sense of self-agency tied to the sense of other-agency.

In order to explain the observed changes in the sense of agency during joint action, researchers proposed various accounts (Seemann, 2009; Pacherie, 2012, 2014; Salmela and Nagatsu, 2017; Salice et al., 2019; Silver et al., 2021; De Vicariis et al., 2022). Firstly, one proposition specifically pertained to hierarchically structured joint actions where some agents take on the role of leaders while others obey. Such situations would entail an expansion of self-agency (Pacherie, 2012), also referred to as a vicarious sense of agency for others’ actions (Silver et al., 2021). In other words, the leader’s self-agentic identity inflates to encompass the actions of others (Figure 2A). Research indeed showed that when a leader’s action triggered the action of a follower, the leader displayed Intentional Binding between her/his action and the action of the follower (Pfister et al., 2014; Capozzi et al., 2016). Conversely, when an action was performed on the request of a leader, Intentional Binding (Caspar et al., 2016, 2018, 2020; Barlas, 2019) and sensory attenuation (Weiss et al., 2011; Caspar et al., 2016, 2018, 2020) decreased for the follower’s self-generated actions.

Secondly, researchers have proposed accounts relevant for egalitarian joint actions, that is, when agents share equal responsibility for the outcome and exert similar degrees of control over the joint action (Obhi and Hall, 2011a; Pacherie, 2014; Salice et al., 2019). It has been suggested that two different agentic states can emerge in this context (Pacherie, 2012, 2014). The first is the sense of shared agency, where self-agency is maintained alongside a broader experience of joint agency - that is, one retains the individual feeling of control (“I’m doing this”) alongside a sense of joint agency (“we are doing this”) (Figure 2B). The second is a merging of each group member’s agency into a single collective agency. In this way, one’s self-agency is dissolved into the collective agency of the group. This phenomenal state has been termed “we-agency” (Pacherie, 2012, 2014) and, in its purest form, entails boundary loss between group members, the feeling they are one and, as a consequence, the disappearance of the sense of self-agency (Figure 2C). The we-agency hypothesis was inspired by anecdotal reports of the agentic experience felt in the context of team sports, musical or dance performance, military drill or even in large audiences present at sports events, concerts or demonstrations (Pacherie, 2014; Loehr, 2022) (here it should be noted that some researchers favor replacing the term “we-agency” by “united agency” (Loehr, 2022), but the principle is the same). The difference between shared and we-agency states depends on the extent to which one retains a sense of self-agency (Silver et al., 2021). Diminishment of self-agency thus characterizes we-agency.

However, despite the widespread adoption of the concept of we-agency, the evidence in support of it is lacking, even in studies purporting to demonstrate the emergence of we-agency. For example, a phenomenological study on members of a music quartet - one of the prototypical contexts in which we-agency emerges - construed the musicians’ descriptions as revealing a shift to we-agency during musical performance (Salice et al., 2019). In the quartet’s description, each musician was absorbed in the group’s activity and there could be changes of direction in the performance that no one planned nor initiated. These changes in agentic experience were interpreted by the authors as illustrating a shift from a self-agency to a we-agency. However, from these descriptions alone we would suggest that there is no evidence of the emergence of we-agency (in which self-agency is supplanted by a collective agentic experience). Rather, one may argue that these descriptions instead speak to a loss of sense of agency, including self and joint agency. Interestingly, the authors appeared to endorse this viewpoint, writing that musicians “experience the music as an agential system whose “will” you must subject yourself to in order to deliver an authentic performance” (p. 14) (Salice et al., 2019).

The we-agency hypothesis has been challenged by some researchers (Salmela and Nagatsu, 2017; Forlè, 2021) who view the dichotomy between shared and we-agency as unwarranted. They suggest that individuals cooperating together in action develop an emotional linkage through mechanisms such as emotional contagion or facial mimicry (Palagi et al., 2020). This linkage enables them to share their sense of agency and the dissolving of individual agentic identities into a collective we-agency seems unnecessary. Understood this way, there need not be a trade-off between the sense of self-agency and that of joint agency; that is, increasing one does not necessarily decrease the other.

Returning to the studies summarized in the previous section on the Intentional Binding effect, they highlighted the existence of a sense of agency for the co-actor’s actions in joint action contexts, yet they did not demonstrate the existence of we-agency *per se*. The Intentional Binding effects they observed could indifferently be indicative of a sense of agency for the self and for the co-actor or a sense of we-agency encompassing the collective as a whole. As the we-agency account posits the emergence of a sense of joint agency at the expense of self-agency, using intentional binding or sensory attenuation to test this hypothesis entails isolating joint action-effects from individual action-effects, which is challenging in the context of a joint action. Explicit measures of the sense of agency may help complement the methodological tools used to address the question of we-agency, provided they can capture nuances of agency states in joint action contexts. One way to experimentally test the we-agency hypothesis is to examine whether the sense of self-agency decreases while joint agency is maintained during joint action. To this end, a study collected explicit judgments of self-agency, other-agency and joint-agency in a setting believed to foster the emergence of we-agency, that is, participants were performing the same movements synchronously thus favoring the blurring of self and others’ agentic identities (Reddish et al., 2020). Participants did indeed feel they had some influence on the other person’s movements and, conversely, that the other person influenced their movements. Yet, ratings of self-agency did not decrease. Additionally, separate studies reported that performing a task with a co-actor had a relatively minor impact on explicit ratings of self-agency compared to completing the same task alone (Dewey et al., 2014) and that ratings of self and joint control correlated with each other (Le Bars et al., 2020). To sum up, the main claim associated with the we-agency hypothesis thus seems to lack experimental support.

The literature on the implicit sense of agency does nevertheless provide some indirect support in favor of the idea that the sense of agency for the self and for the co-actor are tied in joint action. It stems from experimental manipulations leading to a disappearance of the implicit feelings of agency for others as well as for oneself as if they were bound to one another. Several Intentional Binding studies implemented manipulations of this kind by replacing the partner in joint action by a computer or a robot (Obhi and Hall, 2011b; Grynszpan et al., 2019; Sahaï et al., 2019, 2022). The joint action is thus left untouched, but the relationship between co-actors is disrupted. These studies were extremely consistent in revealing that replacing the partner by a robot or computer led to a loss of Intentional Binding for the other’s contribution to joint action - which may be expected - but also for one’s own contribution to action. The latter may be explained by the existence of a link between the agentic identities of the self and partner in joint action. By contrast, the alternate hypothesis of a shared agency, where joint agency is deployed alongside self-agency in joint action (Pacherie, 2012, 2014; Salmela and Nagatsu, 2017) fails to account for the disappearance of the feeling of self-agency, which, accordingly, should never cease to exist. To wrap up, the current state of the literature on egalitarian joint action contexts seems to convey a phenomenology of the implicit sense of agency in which self-agency neither dissolves into a larger we-agency, nor co-exists with a joint agency, but is tied to the feeling of the co-actor’s agency.

Loss of sense of agency for one’s action as well as for the co-actor’s actions has actually also been reported in the context of hierarchically structured joint actions. As mentioned earlier, individuals following the orders of a leader displayed a reduced implicit sense of agency (Weiss et al., 2011; Caspar et al., 2016, 2018, 2020; Barlas, 2019). Interestingly, the available evidence also revealed that, despite feeling agency for the action of the follower (Pfister et al., 2014; Capozzi et al., 2016), leaders failed to feel agency for the joint outcome, that is, the effect of the follower’s action (Pfister et al., 2014; Capozzi et al., 2016; Caspar et al., 2018). The asymmetry of roles, responsibility and potency in hierarchically structured joint actions may hamper the relationship between co-actors, resulting in a loss of self and other agencies as in the examples above involving partnerships between humans and machines.

## 4 Concluding remarks and future directions

The current state of the literature on the sense of agency in joint actions reveals a lack of conceptual clarity, with the use of terms such as joint agency, shared agency and we-agency. Those terms are sometimes employed interchangeably (Loehr, 2022) while other times they denote distinct concepts (Pacherie, 2012, 2014; Silver et al., 2021). As mentioned earlier, there is currently no evidence that self-agency is dissolved into a collective agentic identity as posited by the we-agency conceptualization (Pacherie, 2012, 2014). In light of this, we suggest that the concept of we-agency should be abandoned until proven otherwise. Based on the evidence reviewed above, we recommend a more parsimonious account to describe the sense of agency in joint actions. What is left when discounting the we-agency hypothesis is the idea of joint agentic identities. The terms joint, shared and we-agency would then refer to the same agentic state where co-actors retain a sense of agency for themselves as well as for their co-actors, those two agentic identities being tied to one another (Figure 2E). A more accurate term than those coined until now to characterize this state could be *the sense of joint agencies*, keeping the plural to underscore the co-existence of multiple agentic identities. Such an agentic state emerges in cases of egalitarian joint actions. A loss of the sense of agency for joint action-effects can also occur when the relationship between co-actors is disrupted, as in hierarchically structured groups (Pfister et al., 2014; Capozzi et al., 2016; Barlas, 2019), coercion (Caspar et al., 2016, 2018, 2020) or human-machine interactions (Obhi and Hall, 2011b; Grynszpan et al., 2019; Sahaï et al., 2019, 2022) (Figure 2D).

It is important to emphasize that evidence for the sense of joint agencies predominantly relies on the Intentional Binding effect in the current state of the literature. As stated above, this measure could be usefully complemented by explicit measures. However, as reviewed here, research on the explicit sense of agency is still in the process of refining its methodological tools. A recent study (Reddish et al., 2020) offered an interesting attempt in this direction by formulating questions on the sense of agency that used the first person plural. It should also be noted that, like implicit measures, explicit measures also have their limitations (Moore, 2016). Considering this, we would encourage researchers to adopt multi-method approaches in order to fully understand the broad spectrum of agentic experience in joint action.

The cooperation tasks currently used in lab investigations of the sense of agency limit their potency to create a truly egalitarian joint action. A major hinderance is that there always is a co-actor who initiates the action and another who follows. Though this seems hard to circumvent, researchers should strive to design tasks in which co-actors contribute equally to the joint outcome in terms of decision making, despite one of them starting the action first. Studies in which co-actors’ actions are interdependent (Dewey et al., 2014; Le Bars et al., 2020) offer examples of such tasks. Finally, we advocate for more investigation of joint actions in ecological contexts as in Salice et al.’s (2019) study on live music. Online collaborative work (e.g., text editing) may offer an interesting avenue for such research as it allows logging and controlling interactions, while providing a realistic environment.

## Data availability statement

The original contributions presented in the study are included in the article/supplementary material, further inquiries can be directed to the corresponding author.

## Author contributions

AL: Writing – original draft, Writing – review & editing. JM: Writing – original draft, Writing – review & editing. BB: Writing – original draft, Writing – review & editing. OG: Writing – original draft, Writing – review & editing.

## References

[ref1] Barlas Z. (2019). When robots tell you what to do: sense of agency in human- and robot-guided actions. Conscious. Cogn. 75:102819. doi: 10.1016/j.concog.2019.102819, PMID: 31541968

[ref2] Bolt N. K. Loehr J. D. (2017). The predictability of a partner’s actions modulates the sense of joint agency. Cognition 161, 60–65. doi: 10.1016/j.cognition.2017.01.004, PMID: 28110236

[ref3] Bolt N. K. Loehr J. D. (2021). Sensory attenuation of the auditory P2 differentiates self- from partner-produced sounds during joint action. J. Cogn. Neurosci. 33, 2297–2310. doi: 10.1162/jocn_a_01760, PMID: 34272962

[ref4] Bolt N. K. Poncelet E. M. Schultz B. G. Loehr J. D. (2016). Mutual coordination strengthens the sense of joint agency in cooperative joint action. Conscious. Cogn. 46, 173–187. doi: 10.1016/j.concog.2016.10.001, PMID: 27764684

[ref5] Capozzi F. Becchio C. Garbarini F. Savazzi S. Pia L. (2016). Temporal perception in joint action: this is MY action. Conscious. Cogn. 40, 26–33. doi: 10.1016/j.concog.2015.12.004, PMID: 26741858

[ref6] Caspar E. A. Christensen J. F. Cleeremans A. Haggard P. (2016). Coercion changes the sense of Agency in the Human Brain. Curr. Biol. 26, 585–592. doi: 10.1016/j.cub.2015.12.067, PMID: 26898470 PMC4791480

[ref7] Caspar E. A. Cleeremans A. Haggard P. (2018). Only giving orders? An experimental study of the sense of agency when giving or receiving commands. PLoS One 13:e0204027. doi: 10.1371/journal.pone.0204027, PMID: 30256827 PMC6157880

[ref8] Caspar E. A. Lo Bue S. De Saldanha M. da Gama P. A. Haggard P. Cleeremans A. (2020). The effect of military training on the sense of agency and outcome processing. Nat. Commun. 11:4366. doi: 10.1038/s41467-020-18152-x, PMID: 32868764 PMC7459288

[ref9] Cho P. S. Escoffier N. Mao Y. Green C. Davis R. C. (2020). Beyond physical entrainment: competitive and cooperative mental stances during identical joint-action tasks differently affect inter-subjective neural synchrony and judgments of agency. Soc. Neurosci. 15, 368–379. doi: 10.1080/17470919.2020.1727949, PMID: 32031918

[ref10] De Vicariis C. Chackochan V. T. Sanguineti V. (2022). Game theory and partner representation in joint action: toward a computational theory of joint agency. Phenomenol. Cogn. Sci. doi: 10.1007/s11097-022-09819-5

[ref11] Dewey J. A. Pacherie E. Knoblich G. (2014). The phenomenology of controlling a moving object with another person. Cognition 132, 383–397. doi: 10.1016/j.cognition.2014.05.002, PMID: 24879353

[ref12] Forlè F. (2021). The sense of we-agency and vitality attunement: between rhythmic alignment and emotional attunement. Phenomenol. Cogn. Sci. doi: 10.1007/s11097-021-09779-2

[ref13] Frith C. D. (2014). Action, agency and responsibility. Neuropsychologia 55, 137–142. doi: 10.1016/j.neuropsychologia.2013.09.007 24036357

[ref14] Grynszpan O. Sahaï A. Hamidi N. Pacherie E. Berberian B. Roche L.. (2019). The sense of agency in human-human vs human-robot joint action. Conscious. Cogn. 75:102820. doi: 10.1016/j.concog.2019.102820, PMID: 31561189

[ref15] Haggard P. (2017). Sense of agency in the human brain. Nat. Rev. Neurosci. 18, 196–207. doi: 10.1038/nrn.2017.14 28251993

[ref16] Haggard P. Chambon V. (2012). Sense of agency. Curr. Biol. 22, R390–R392. doi: 10.1016/j.cub.2012.02.040 22625851

[ref17] Hayashida K. Nishi Y. Osumi M. Nobusako S. Morioka S. (2021). Goal sharing with others modulates the sense of agency and motor accuracy in social contexts. PLoS One 16:e0246561. doi: 10.1371/journal.pone.0246561, PMID: 33539426 PMC7861436

[ref18] Jenkins M. Esemezie O. Lee V. Mensingh M. Nagales K. Obhi S. S. (2021). An investigation of “we” agency in co-operative joint actions. Psychol. Res. 85, 3167–3181. doi: 10.1007/s00426-020-01462-6, PMID: 33398449

[ref19] Le Bars S. Devaux A. Nevidal T. Chambon V. Pacherie E. (2020). Agents’ pivotality and reward fairness modulate sense of agency in cooperative joint action. Cognition 195:104117. doi: 10.1016/j.cognition.2019.104117, PMID: 31751814

[ref20] Le Besnerais A. Prigent E. Grynszpan O. (2023). Agency and social affordance shape visual perception. Cognition 233:105361. doi: 10.1016/j.cognition.2022.105361, PMID: 36563643

[ref21] Loehr J. D. (2013). Sensory attenuation for jointly produced action effects. Front. Psychol. 4:172. doi: 10.3389/fpsyg.2013.00172, PMID: 23596429 PMC3622880

[ref22] Loehr J. D. (2018). Shared credit for shared success: successful joint performance strengthens the sense of joint agency. Conscious. Cogn. 66, 79–90. doi: 10.1016/j.concog.2018.11.001, PMID: 30445276

[ref23] Loehr J. D. (2022). The sense of agency in joint action: an integrative review. Psychon. Bull. Rev. 29, 1089–1117. doi: 10.3758/s13423-021-02051-3, PMID: 35146702

[ref24] McNeese N. J. Demir M. Cooke N. J. Myers C. (2018). Teaming with a synthetic teammate: insights into human-autonomy teaming. Hum. Factors 60, 262–273. doi: 10.1177/0018720817743223 29185818

[ref25] Moore J. W. (2016). What is the sense of agency and why does it matter? Front. Psychol. 7:1272. doi: 10.3389/fpsyg.2016.01272, PMID: 27621713 PMC5002400

[ref26] Moore J. W. Obhi S. S. (2012). Intentional binding and the sense of agency: a review. Conscious. Cogn. 21, 546–561. doi: 10.1016/j.concog.2011.12.002, PMID: 22240158

[ref27] Obhi S. S. Hall P. (2011a). Sense of agency and intentional binding in joint action. Exp. Brain Res. 211, 655–662. doi: 10.1007/s00221-011-2675-2, PMID: 21503647

[ref28] Obhi S. S. Hall P. (2011b). Sense of agency in joint action: influence of human and computer co-actors. Exp. Brain Res. 211, 663–670. doi: 10.1007/s00221-011-2662-7, PMID: 21503652

[ref29] Pacherie E. (2012). The phenomenology of joint action: self-agency vs. joint-agency. In: Seemann A. (Éd.), Joint attention: New developments (p. 343–389). MIT Press.

[ref30] Pacherie E. (2014). How does it feel to act together? Phenomenol. Cogn. Sci. 13, 25–46. doi: 10.1007/s11097-013-9329-8

[ref31] Palagi E. Celeghin A. Tamietto M. Winkielman P. Norscia I. (2020). The neuroethology of spontaneous mimicry and emotional contagion in human and non-human animals. Neurosci. Biobehav. Rev. 111, 149–165. doi: 10.1016/j.neubiorev.2020.01.020 31972204

[ref32] Pfister R. Obhi S. S. Rieger M. Wenke D. (2014). Action and perception in social contexts: intentional binding for social action effects. Front. Hum. Neurosci. 8:667. doi: 10.3389/fnhum.2014.00667, PMID: 25228869 PMC4151154

[ref33] Poonian S. K. Cunnington R. (2013). Intentional binding in self-made and observed actions. Exp. Brain Res. 229, 419–427. doi: 10.1007/s00221-013-3505-5, PMID: 23575956

[ref34] Reddish P. Tong E. M. W. Jong J. Whitehouse H. (2020). Interpersonal synchrony affects performers’ sense of agency. Self Identity 19, 389–411. doi: 10.1080/15298868.2019.1604427

[ref35] Roselli C. Ciardo F. Wykowska A. (2021). Intentions with actions: the role of intentionality attribution on the vicarious sense of agency in human–robot interaction. Q. J. Exp. Psychol. 75, 616–632. doi: 10.1177/17470218211042003 34472397

[ref36] Sahaï A. Caspar E. De Beir A. Grynszpan O. Pacherie E. Berberian B. (2022). Modulations of one’s sense of agency during human–machine interactions: a behavioural study using a full humanoid robot. Q. J. Exp. Psychol. 76, 606–620. doi: 10.1177/17470218221095841, PMID: 35400221

[ref37] Sahaï A. Desantis A. Grynszpan O. Pacherie E. Berberian B. (2019). Action co-representation and the sense of agency during a joint Simon task: comparing human and machine co-agents. Conscious. Cogn. 67, 44–55. doi: 10.1016/j.concog.2018.11.008, PMID: 30522081

[ref38] Salice A. Høffding S. Gallagher S. (2019). Putting plural self-awareness into practice: the phenomenology of expert musicianship. Topoi 38, 197–209. doi: 10.1007/s11245-017-9451-2

[ref39] Salmela M. Nagatsu M. (2017). How does it really feel to act together? Shared emotions and the phenomenology of we-agency. Phenomenol. Cogn. Sci. 16, 449–470. doi: 10.1007/s11097-016-9465-z

[ref40] Sebanz N. Bekkering H. Knoblich G. (2006). Joint action: bodies and minds moving together. Trends Cogn. Sci. 10, 70–76. doi: 10.1016/j.tics.2005.12.009, PMID: 16406326

[ref41] Seemann A. (2009). Joint agency: Intersubjectivity, sense of control, and the feeling of trust. Inquiry 52, 500–515. doi: 10.1080/00201740903302634

[ref42] Shiraishi M. Shimada S. (2021). Inter-brain synchronization during a cooperative task reflects the sense of joint agency. Neuropsychologia 154:107770. doi: 10.1016/j.neuropsychologia.2021.107770 33548249

[ref43] Silver C. A. Tatler B. W. Chakravarthi R. Timmermans B. (2021). Social agency as a continuum. Psychon. Bull. Rev. 28, 434–453. doi: 10.3758/s13423-020-01845-1, PMID: 33289061 PMC8062427

[ref44] Suzuki K. Lush P. Seth A. K. Roseboom W. (2019). Intentional binding without intentional action. Psychol. Sci. 30, 842–853. doi: 10.1177/0956797619842191 31023161

[ref45] Synofzik M. Vosgerau G. Newen A. (2008). Beyond the comparator model: a multifactorial two-step account of agency. Conscious. Cogn. 17, 219–239. doi: 10.1016/j.concog.2007.03.010, PMID: 17482480

[ref46] van der Wel R. P. R. D. (2015). Me and we: metacognition and performance evaluation of joint actions. Cognition 140, 49–59. doi: 10.1016/j.cognition.2015.03.011, PMID: 25880341

[ref47] van der Wel R. P. R. D. Sebanz N. Knoblich G. (2012). The sense of agency during skill learning in individuals and dyads. Conscious. Cogn. 21, 1267–1279. doi: 10.1016/j.concog.2012.04.001, PMID: 22541646

[ref48] Weiss C. Herwig A. Schütz-Bosbach S. (2011). The self in social interactions: sensory attenuation of auditory action effects is stronger in interactions with others. PLoS One 6:e22723. doi: 10.1371/journal.pone.0022723, PMID: 21818373 PMC3144943

[ref49] Wynne K. T. Lyons J. B. (2018). An integrative model of autonomous agent teammate-likeness. Theor. Issues Ergon. Sci. 19, 353–374. doi: 10.1080/1463922X.2016.1260181

[ref50] Zapparoli L. Paulesu E. Mariano M. Ravani A. Sacheli L. M. (2022). The sense of agency in joint actions: a theory-driven meta-analysis. Cortex 148, 99–120. doi: 10.1016/j.cortex.2022.01.002, PMID: 35168155

